# Adipose-Derived Stem Cells Based on Electrospun Biomimetic Scaffold Mediated Endothelial Differentiation Facilitating Regeneration and Repair of Abdominal Wall Defects *via* HIF-1α/VEGF Pathway

**DOI:** 10.3389/fbioe.2021.676409

**Published:** 2021-07-07

**Authors:** Wenpei Dong, Zhicheng Song, Suihong Liu, Ping Yu, Zhipeng Shen, Jianjun Yang, Dongchao Yang, Qinxi Hu, Haiguang Zhang, Yan Gu

**Affiliations:** ^1^Department of General Surgery, Hernia and Abdominal Wall Surgery Center of Shanghai Jiao Tong University, Shanghai Ninth People’s Hospital, Shanghai Jiao Tong University School of Medicine, Shanghai, China; ^2^Rapid Manufacturing Engineering Center, Shanghai University, Shanghai, China; ^3^Department of Pharmacy, Ruijin Hospital, Shanghai Jiao Tong University School of Medicine, Shanghai, China

**Keywords:** abdominal wall defects, biomimetic scaffold, tissue engineering, adipose-derived stem cells, endothelial differentiation, angiogenesis

## Abstract

Application of synthetic or biological meshes is the main therapy for the repair and reconstruction of abdominal wall defects, a common disease in surgery. Currently, no ideal materials are available, and there is an urgent need to find appropriate ones to satisfy clinical needs. Electrospun scaffolds have drawn attention in soft tissue reconstruction. In this study, we developed a novel method to fabricate a composite electrospun scaffold using a thermoresponsive hydrogel, poly (*N*-isopropylacrylamide)-block-poly (ethylene glycol), and a biodegradable polymer, polylactic acid (PLA). This scaffold provided not only a high surface area/volume ratio and a three-dimensional fibrous matrix but also high biocompatibility and sufficient mechanical strength, and could simulate the native extracellular matrix and accelerate cell adhesion and proliferation. Furthermore, rat adipose-derived stem cells (ADSCs) were seeded in the composite electrospun scaffold to enhance the defect repair and regeneration by directionally inducing ADSCs into endothelial cells. In addition, we found early vascularization in the process was regulated by the hypoxia inducible factor-1α (HIF-1α)/vascular endothelial growth factor (VEGF) pathway. In our study, overexpression of HIF-1α/VEGF in ADSCs using a lentivirus system promoted early vascularization in the electrospun scaffolds. Overall, we expect our composite biomimetic scaffold method will be applicable and useful in abdominal wall defect regeneration and repair in the future.

## Introduction

The abdominal wall has a significant protective effect on abdominal organs. Etiologic factors, including abdominal trauma, lead to abdominal wall defects. At present, synthetic or biological meshes are applied in the repair and reconstruction of abdominal defects (Song et al., 2018). However, the implanted meshes, particularly synthetic non-absorbable ones, may stimulate and erode the surrounding tissues as foreign bodies, which can cause numerous complications. Therefore, to repair abdominal wall defects, there is an urgent need to find ideal materials possessing sufficient strength, minimized immune inflammatory response, early vascularization, and angiogenesis (Shokry et al., 2019).

Currently, scaffolds fabricated by the electrospinning method have gained prominence for soft tissue reconstruction (Liu et al., 2015; Mori da Cunha et al., 2019; Kaya et al., 2020). Electrospun scaffolds have the advantages of light weight, high surface area/volume ratio, and a three-dimensional (3D) fibrous matrix with interconnected pores, which mimic the native extracellular matrix (James and Laurencin, 2016; Pensa et al., 2019; Kaya et al., 2020). Poly (N-isopropylacrylamide) (PNIPAAm), a thermoresponsive hydrogel, self-assembles at the lower critical solution temperature (LCST) of 32°C without a chemical crosslinking agent and is extensively applied in drug-controlled release and tissue engineering (Donderwinkel et al., 2017; Suntornnond et al., 2017; Bordat et al., 2018). It is reported that cell adherence and proliferation are favored when culturing in a 3D medium fabricated using PNIPAAm (Suntornnond et al., 2017). However, insufficient mechanical strength, non-degradability, and slow temperature response limit its application. Therefore, a block polymer of PNIPAAm, named as poly (N-isopropylacrylamide)-block-poly(ethylene glycol) (PNIPAAm-b-PEG), was synthesized, which has a more rapid temperature response, lower LCST, and improved degradation capability when retaining the advantages of PNIPAAm (Pollock and Healy, 2010). However, electrospun scaffolds have comparatively weak mechanical characteristics of low strength and stiffness, which limit their application in the repair of abdominal wall defects for which a relatively high mechanical strength is needed (Khorshidi et al., 2016). To overcome this disadvantage, polylactic acid (PLA), a biodegradable polymer of lactic acid, has been reported to be applied in tissue engineering scaffolds for providing sufficient mechanical and tensile strength (Dinh Nguyen et al., 2018; Singhvi et al., 2019).

Early vascularization is the premise of abdominal wall defect repair and regeneration, whereas abdominal wall-implanted scaffolds need rapid vascularization to realize tissue regeneration because of the limited diffusion distance (150–200 μm) of oxygen *in vivo* (Staubli et al., 2017). Therefore, promoting early vascularization of implant materials is the key target in abdominal wall tissue engineering. Adipose-derived stem cells (ADSCs) have the potential to multi-differentiate into adipocytes, osteoblasts, and endothelial cells (Naderi et al., 2017). Recent studies have demonstrated the repair and regeneration ability of ADSCs at both cellular and histological levels (Shingyochi et al., 2015). Methods such as adding exogenous cytokines, pretreatment of platelet-rich plasma, etc., have been tested to induce the endothelial differentiation of ADSCs for tissue defect repair. However, their differentiating effect was restricted by the non-uniform distribution of cytokines, short half-life of the biological activity, difficult control release and other problems (Volz et al., 2016; Ma et al., 2017; Chen and Liao, 2018). As an important inducing factor, vascular endothelial growth factor (VEGF) plays a key role in stem cell-based regenerative therapies in endothelial differentiation (Rodrigues et al., 2010). Enhanced secretion of VEGF in ADSCs using gene transfection technology can stimulate angiogenesis of soft tissue wounds, bone defects, and ischemic hindlimbs (Nauta et al., 2013; Shevchenko et al., 2013; Kang et al., 2017). Furthermore, another transcription factor, hypoxia inducible factor-1α (HIF-1α), which is affected by oxygen concentration, can regulate the cytokine secretion of ADSCs (Lang et al., 2019). Overexpression of HIF-1α improves the vascularized effect of stem cells, whereas silencing HIF-1α causes continuous wound non-healing (Hong et al., 2014). HIF-1α regulates the expression of downstream target genes, such as VEGF and basic fibroblast growth factor, to promote host cell migration, adhesion, and cytokine secretion, stimulating angiogenesis in wound repair (Ruthenborg et al., 2014). That revealed angiogenesis in the implanted scaffold could be achieved by inducing ADSCs endothelial differentiation *via* regulation of HIF-1α/VEGF pathway.

In this study, a biocompatible scaffold consisting of PNIPAAm-b-PEG/PLA was electrospun, and concurrently the properties of the scaffold were characterized. Isolated ADSCs were cultured and seeded in the scaffold after transfection by lenti-HIF-1α or lenti-VEGF, to confirm the regulation of the HIF-1α/VEGF pathway for directionally inducing ADSCs into endothelial cells to promote the early vascularization in abdominal wall reconstruction.

## Materials and Methods

### Isolation, Culture, and Characterization of ADSCs

Sprague–Dawley (SD) rats (males, 4–6 weeks old) were fed *ad libitum* on a standard diet and kept on a 12-h-light/12-h-dark cycle at room temperature of 22°C according to the Guide for the Care and Use of Laboratory Animals (United States National Institutes of Health Publication). All the experiments were reviewed and approved by the Animal Experimental Ethics Committee of Shanghai Ninth People’s Hospital affiliated to the Shanghai Jiao Tong University School of Medicine (Certificate No.SH9H-2019-A475-1). The protocol for ADSCs isolation and culture was based on previously published study (Han et al., 2015). ADSCs were obtained for use in experiments after the third passage. To characterize the ADSCs, flow cytometry was performed to detect cell markers of CD29, CD31, CD44H, CD45, and CD90 (Biolegend, United States), adipogenic, and osteogenic differentiation capacities were also confirmed (Minteer et al., 2013; Han et al., 2015).

### Fabrication of Biomimetic Scaffold

To obtain the biomimetic scaffold, a 3D printing-assisted electrospinning system (Rapid Manufacturing Engineering Center, Shanghai University, China) was used. The physical blending PNIPAAm-b-PEG (9 kD, RuiXi Biological Technology, China)/PLA (60 kD, Aladdin, China) composite solution was delivered using a 20-gauge needle. A 160-μm internal diameter needle was used and mounted on the electrospinning system. The nanofibers were spun at a fixed electrical potential of 15 kV across the distance of 15 cm between the needle tip and the receiving platform. The electrospinning process was completed in 4 h, forming a nanofiber membrane on the receiving platform. Finally, the electrospun nanofiber membrane was torn and placed in an oven for 3 h for further evaporation and drying at 37°C. In addition, biomimetic scaffolds were fabricated with different weight ratios of PNIPAAm-b-PEG and PLA (1:3, 1:4, 1:5, 1:6, 1:7, 1:9, and 0:1).

### Characterization of Biomimetic Scaffold

The mechanical properties, equilibrium water content (EWC), water contact angle, and degradation rate were tested to evaluate the characteristics of the biomimetic scaffold. There were eight groups in this part which were polypropylene mesh (PP) group, PLA group, and the rest different ratio of PNIPAAm-b-PEG/PLA (1:3, 1:4, 1:5, 1:6, 1:7, and 1:9) groups.

The mechanical properties were assessed at room temperature using a uniaxial material testing machine (WDW-1, Songdun Machine Equipment Co., Ltd., China). Scaffold samples were cut into 20 × 15 mm^2^ dimensions and stretched along their longitudinal axes at a cross-head speed of 5 mm/min until fracture. The stiffness (N/mm) was determined by calculating the slope of the load (linear portion) vs. displacement plot for five samples in each group.

The EWC of a scaffold was defined as the water content of the swollen scaffold. The wet weight (Ww) of each sample was recorded after immersion in PBS at 37°C for 24 h followed by careful removal of the surface liquid. The dry weight (Wd) was recorded as previously described (Liu et al., 2008). The EWC was calculated using the following equation:

(1) $$\text{EWC}=\frac{\text{Ww}-\text{Wd}}{\text{Ww}}\times 100\%$$

To analyze the hydrophilicity of the samples, the water contact angle was measured by an OCA 15EC optical contact angle measuring and contour analysis system (DataPhysics Instruments GmbH, Germany) at room temperature using the sessile drop method. Briefly, a droplet of MilliQ water was captured with a CCD camera after standing for 30 s on the surface of a sample. The water contact angle was determined as the average of at least six different collected measurements. However, PP group could not receive this evaluation for its macroporous and reticular structure.

The degradation rate of each scaffold sample (20 × 15 mm^2^) was examined based on the relative mass loss at 37°C in PBS. After weighing the original dried sample (Wo), it was immersed and incubated for 4, 8, and 12 weeks. Subsequently, it was rinsed gently to remove any attachment/precipitate and weighed again after drying in an oven/vacuum at 40°C for 24 h. The final mass of the dries scaffold was recorded (Wt). The *in vitro* degradation rate was calculated as follows:

(2) $$\mathrm{Weight}\mathrm{loss}\left(\%\right)=\frac{\text{Wo}-\mathrm{Wt}}{\text{Wo}}\times 100\%$$

### Biocompatible Test of Biomimetic Scaffold

To validate the toxic effect of synthetic PNIPAAm-b-PEG, cell counting kit-8 (CCK-8; Dojindo, Japan) was used. Firstly, PNIPAAm-b-PEG was sufficiently dissolved in complete medium for different concentrations (10^–4^, 10^–3^, 10^–2^, 10^–1^, and 1 mM). Then, cell experiment was divided into six groups: the control group (cells cultured in complete medium) and the five intervention groups (cells cultured in different concentrations of PNIPAAm-b-PEG). Cell viability was detected after 1, 3, and 7 days culture. The optical density (OD) values were obtained at a wavelength of 450 nm by a Synergy HT spectrophotometer (BioTek Instruments, United States). The cytotoxicity of the scaffolds was tested using a live/dead viability assay kit (Life Technologies Inc., China). The scaffold samples were placed in 12-well plates and soaked overnight in the complete medium. ADSCs were seeded on the scaffolds at a density of 5 × 10^4^ cells/well per 1.5 mL. After 5 days of incubation, the live/dead viability assay kit was used to assess the cell viability. The live cells were dyed with calcein AM (green), and the dead cell nuclei with propidium iodide (PI, red), which were subsequently observed by an inverted fluorescence microscope (Ti-s; Nikon, Japan). Furthermore, the seeded scaffolds were rinsed in PBS, fixed with 2.5% glutaraldehyde overnight at 4°C, and dehydrated in a graded series of alcohols at 37°C. After air drying and gold coating, the samples were further observed using a scanning electron microscope (SU1510; Hitachi, Japan).

### Hypoxic Culture of ADSCs

Adipose-derived stem cells were cultured in a hypoxic environment which simulated the hypoxia in the early period of abdominal wall defect area. The method was based on previously published (Bigot et al., 2015). The hypoxic ADSCs were cultured in an anoxic incubator containing 1% O_2_, 5% CO_2_, and 94% N_2_ at 37°C, while the control cells were cultured in a normoxic incubator. Expression of HIF-1α, VEGF, and α-SMA were measured by western blotting, and RT-qPCR testing after 21 days’ culture.

### Lentiviral and Small Interfering RNA (siRNA) Transfection of ADSCs

HIF-1α and VEGF overexpression models were created by lentiviral transfection. One day before cell transfection, ADSCs were seeded and incubated in 60-mm cell dishes at a density of 1 × 10^5^ cells/dish. Lentivirus kits targeting HIF-1α and VEGF (GeneChem, China) were used at a multiplicity of infection of 50, which was ensured in a preliminary experiment. The infected ADSCs were divided into control group (blank vector), lenti-HIF-1α, and lenti-VEGF groups. The fresh culture medium was changed after 16 h of transfection. Cells were observed after 72 h, and the overexpression efficiency was assessed by the reverse transcript-quantitative polymerase chain reaction (RT-qPCR) test and western blotting. After continuous culturing to the fifth passage, ADSCs were digested, transferred, and seeded in the scaffolds for use.

HIF-1α knockdown was performed using siRNA. Cells were divided into four groups: a negative control group (transfected with negative control siRNA) and three si-HIF-1α groups (transfected with three sequences of HIF-1α siRNA). Chemically synthesized siRNAs (50 nM/well; Sangon Biotech, China) were transfected into the cultured ADSCs with Lipofectamine 3000 (Invitrogen, United States) in six-well culture plates according to the instructions of the manufacturer for 6 h and subsequently provided with new medium. Cells were harvested to isolate the total RNA and protein 48 h after the transfection. The HIF-1α siRNA sequences were sense 1, 5′-GCUCACCAUCAGU UACUUATT-3′; antisense 1, 5′-UAAGUAACUGAUGGUGAGC CT-3′; sense 2, 5′-CCAUGUGACCAUGAGGAAATT-3′; anti sense 2, 5′-UUUCCUCAUGGUCACAUGGAT-3′; sense 3, 5′-GCCAGCAAGUCCUUCUGAUTT-3′; antisense 3, 5′-AUCAG AAGGACUUGCUGGCTG-3′, and negative control siRNA (sense, 5′-UUCUCCGAACGUGUCACGUTT-3′; antisense, 5′-ACGUGACACGUUCGGAGAATT-3′).

### Transmission Electron Microscopy

Samples were prepared by collecting ADSCs transfected by lenti-HIF-1α and lenti-VEGF. These two kinds of transfected ADSCs were cultured for 21 days. TEM images were acquired using a Hitachi TEM system (HT7700; Hitachi, Japan) to detect whether Weibel-Palade bodies (WPBs), an endothelial cell marker, existed (Amerion et al., 2018).

### Regeneration and Repair of Abdominal Wall Defect in a Rat Model

Abdominal wall defect was created in a rat model as described previously (*n* = 6 per group) (Yang et al., 2018). There were seven groups:(1) the abdominal wall defect group reconstructed by PP mesh; (2) the group repaired by PLA scaffold; (3) the group repaired by the best ratio of PNIPAAm-b-PEG/PLA scaffold; (4–7) the group reconstructed by PNIPAAm-b-PEG/PLA scaffold with ADSCs, blank vector transfected, lenti-HIF-1α, or lenti-VEGF transfected ADSCs. Briefly, female rats (200 ± 20 g) were anaesthetized by intraperitoneal injection of pentobarbital sodium (30 mg/kg). A longitudinal midline skin incision (4 cm) was made to expose the surgical area, and a full-thickness abdominal wall defect (2 × 2 cm^2^), including the fascia, rectus abdominis, and peritoneum, was created. Scaffold was firstly put on 100 mm dish. Then 3 mL complete medium was added to immerse the scaffold. ADSCs and transfected ADSCs suspension were prepared at a density of 1 × 10^6^ cells/mL. Next, the scaffold gently seeded with 1 mL cell suspension was cultured in a normoxic incubator containing 5% CO_2_ at 37°C. 6 mL complete medium was supplemented 3 h later. The scaffold was cultured overnight before being placed intra-abdominally with a 0.3 cm overlap and fixed tension-free to the abdominal wall with an interrupted 4-0 silk suture (Tianqing Biological Material, China). The skin incision was closed with absorbable 3-0 Vicryl sutures (Ethicon, United States), and the rats were allowed to recover normally. The rats were examined for local and systemic complications, such as wound infection, incision dehiscence, recurrent hernia, and death, every week. They were sacrificed at 4, 8, and 12 weeks. Subsequently, abdominal adhesion was evaluated using the criteria (Supplementary Table 1; Poehnert et al., 2015). The regenerative tissues of the abdominal wall defects were also harvested for further histological and immunohistochemical analyses, western blotting, and RT-qPCR testing.

### Histological and Immunohistochemical Analyses

The regenerated tissues of the abdominal wall defects were fixed with 4% paraformaldehyde, for the histological and immunochemical analyses. Sections of 5 μm in size were used to evaluate the regenerative and fibrotic changes, which were stained using hematoxylin-eosin (H&E) and Masson trichrome staining. To detect the endothelialization and vascularization, the expression of CD34 (1:100; GB111693; Servicebio, China) and α-SMA (1:100; ab7817; Abcam, United Kingdom) were evaluated by immunohistochemical staining. Images were acquired by an inverted fluorescent microscope (Ti-s; Nikon, Japan).

### Detection of Immunofluorescent Staining and Fluorescence *in situ* Hybridization

Tissue sections were used to assess the expression of HIF-1α and VEGF by immunofluorescent staining. The primary antibodies used were anti-HIF-1α (1:50; NB100-105; Novus, United States) and VEGF (1:100; ab1316; Abcam, United Kingdom). To track the endotheliocytes derived from ADSCs, CD34 and rat chromosome Y paint probes (D-1522-050-OR; Metasystems, Germany) were used in fluorescence *in situ* hybridization (FISH) staining. Images were captured using the Nikon Ti-s inverted fluorescent microscope. The immunofluorescence intensity results of HIF-1α and VEGF were analyzed using ImageJ software (National Institutes of Health, United States).

### Measurement of Western Blot and Semiquantitative RT-qPCR

All the proteins were isolated from the regenerative tissues. After SDS-PAGE and protein transfer, polyvinylidene fluoride membranes were incubated with HIF-1α (1:1,000), VEGF (1:1,000), and α-SMA (1:1,000) antibodies overnight at 4°C, followed by incubation with immunoglobulin-conjugated anti-mouse, IgG (1:5,000; IRDye^®^ 680RD; Li-cor, United States) for 1 h at room temperature. Bands were visualized by an Odyssey CLx imaging system (Li-cor, United States). RT-qPCR test was performed to analyze the mRNA expression of HIF-1α, VEGF, and α-SMA. Total RNA from the regenerative tissues and cultured cells was extracted by an RNA extraction kit (Code No.9767; Takara, Japan) according to the instructions of the manufacturer. Subsequently, cDNA was synthesized using the Superscript First-Strand Synthesis System (Code No. RR047, Takara, Japan), followed by PCR amplification and quantification by performing a SYBR green quantitative PCR reaction on a Quant Studio 6 Flex RT-PCR System (Thermo, United States). The targeted primer sequences were as follows: β-actin forward 5′-ATCACTATCGGCAATGAGCGGTTC-3′ and reverse 5′-CTCCTGCTTGCTGATCCACATCTG-3′; HIF-1α forward 5′-ACATCAAGTCAGCAACGTGGAAGG-3′ and reverse 5′-GCAAGCATCCTGTACTGTCCTGTG-3′; VEGF forward 5′-CGTCGGAGAGCAACGTCACTATG-3′ and reverse 5′-CCGCCTTGGCTT GTCACATCTG-3′; α-SMA forward 5′-AGAACACGGCATCATCACCAACTG-3′ and reverse 5′-TGAGT CACGCCATCTCCAGAGTC-3′. The expression levels of the genes relative to β-actin were calculated by the 2^–ΔΔ*Ct*^ method. The tests were repeated thrice independently.

### Statistical Analysis

All the statistical analyses were performed using SPSS 18.0 (IBM Corp, Armonk, NY, United States) and GraphPad Prism 6 (GraphPad, Inc., La Jolla, CA, United States). The results were expressed as mean ± standard deviation (SD). One-way analysis of variance (ANOVA) with Tukey’s HSD comparison test was conducted to test for significant differences. *P* < 0.05 was considered to be statistically significant.

## Results

### Identification of ADSCs

Initially, for the study, ADSCs isolated from the adipose tissues of male SD rats were cultured and passaged to the third generation and then identified by testing the cell phenotype and differentiation potential (Supplementary Figure 1). Flow cytometry results showed high expression of positive stem cell markers, including CD29 (99.8%), CD44H (98.5%), and CD90 (92.3%), and low expression of negative markers, such as CD31 (0.61%) and CD45 (0.44%) (Supplementary Figure 1A). In addition, the adipogenic and osteogenic differentiation potentials were observed with numerous red lipid droplets and calcium deposits intracellularly (Supplementary Figure 1B).

### Characterization of PNIPAAm-b-PEG/PLA Scaffold

**FIGURE 1:**
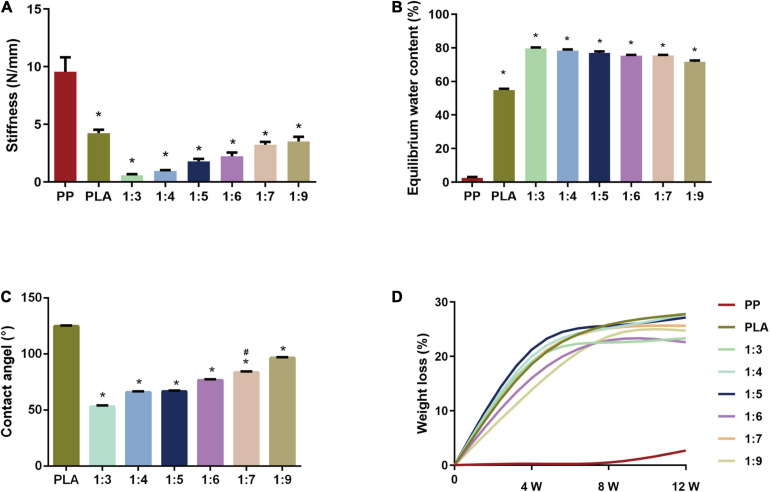
Evaluation of characteristics of PNIPAAm-b-PEG/PLA biomimetic scaffolds. **(A)** Stiffness of scaffolds. *P < 0.05 vs. the PP group. **(B)** Equilibrium water content (EWC) at 37°C in PBS (pH 7.4). *P < 0.05 vs. the PP group. **(C)** Water contact angle of the electropsun scaffolds. *P < 0.05 vs. the PLA group, *#P* < 0.05 vs. the 1:9 group. **(D)** Degradation rate of scaffold samples.

We found the stiffness of the biomimetic scaffold increased when ascending the weight ratio of PLA, reaching a maximum value of 4.248 ± 0.624 N/mm in PLA (the 0:1 PNIPAAm-b-PEG/PLA) scaffold (Figure 1A) in the mechanical testing. The stiffness of the electrospun scaffolds were weaker than that of PP mesh when the latter had a high strength of 9.560 ± 2.808 N/mm. In the PNIPAAm-b-PEG/PLA groups, the mean stiffness at ratios 1:3, 1:4, and 1:5 were lower than 1.6 N/mm, the minimum physiological stiffness of the abdominal wall. Stiffness of PNIPAAm-b-PEG/PLA scaffolds in ratios of 1:7 (3.225 ± 0.559 N/mm), 1:9 (3.514 ± 0.897 N/mm), and the PLA electrospun mesh (4.248 ± 0.624 N/mm) were larger than the requirement of considering extreme behaviors (2.0 N/mm) which indicated the PLA, 1:7, and 1:9 scaffolds satisfied the requirement of abdominal wall stiffness *in vivo*.

The result of EWC in Figure 1B showed PP mesh had the lowest EWC of 2.543 ± 1.329%. The EWC of PNIPAAm-b-PEG/PLA scaffolds had an increasing trend with the raised mass ratio of PNIPAAm-b-PEG. PNIPAAm-b-PEG/PLA meshes in other groups had a higher water content of over 70% than the PLA one (54.852 ± 1.790%). However, there was no significance between 1:7 and 1:9 group.

In the measurement of water contact angle, PP group could not receive this evaluation for its macroporous and reticular structure. The water contact angle of the electrospun scaffolds ranged from 54° to 125° (Figure 1C). The angles in the 1:9 and PLA groups were 96.9 ± 0.7° and 124.3 ± 1.0°, respectively, suggesting hydrophobic surfaces. The surface of the 1:7 scaffold was hydrophilic, with an angle of 84.1 ± 0.5°, which was significantly lower than that of the 1:9 and PLA group, but higher than those of 1:3, 1:4, 1:5, and 1:6 groups with a higher proportion of PNIPAAm-b-PEG.

Degradability assessment of these samples was conducted by the weight loss rate in a period of 12 weeks, as shown in Figure 1D. The weight loss of the electrospun meshes in each group increased with the immersion time, whereas the PP mesh showed almost no mass loss after 12 weeks. Though samples with larger proportions of PNIPAAm-b-PEG seemed to degrade fast, no significant difference was found among these PNIPAAm-b-PEG/PLA scaffolds.

The results above indicated the electrospun scaffolds at ratio 1:7 could meet the requirement with suitable stiffness, EWC, water contact angle and degradability.

### High Biocompatibility of PNIPAAm-b-PEG/PLA Scaffold Promoted ADSCs Proliferation and Spreading

**FIGURE 2:**
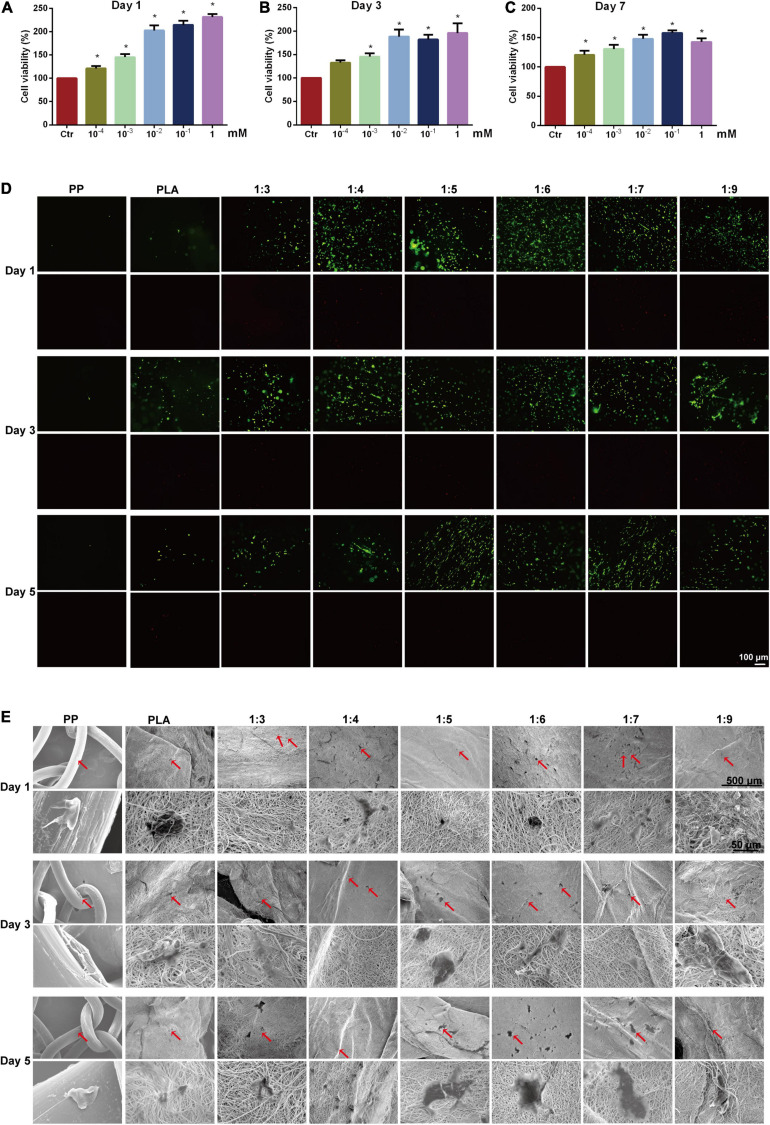
Biocompatibility test of biomimetic scaffolds. Cytotoxicity of PNIPAAm-b-PEG of different concentrations were detected by CCK-8 method on **(A)** days 1, **(B)** 3, and **(C)** 7. **(D)** Cell viability was assessed by live/dead staining of ADSCs after culture of 5 days, in which live cells were stained in green and dead cells were stained in red (Scale bar = 100 μm). **(E)** Cellular morphology of ADSCs (in red arrows) seeded in scaffolds was observed *via* scanning electron microscopy after 1, 3, and 5 days of culture. *P < 0.05 vs. the control group.

As shown in Figures 2A–C, the ADSCs viability was significantly increased after being cultured in the medium containing different concentration of PNIPAAm-b-PEG compared to the control group at day 1, day 3, and day 7. However, there was no significant difference among the three groups (10^–2^, 10^–1^, and 1 mM). The results showed PNIPAAm-b-PEG had little toxicity to ADSCs from 10^–4^ to 1 mM. Appropriate concentration of PNIPAAm-b-PEG could facilitate cell proliferation and viability, whereas higher concentration (10^–1^ and 1 mM) of PNIPAAm-b-PEG did not bring a better benefit than 10^–2^ mM.

Furthermore, the cytocompatible test of the scaffold using a live/dead staining kit showed almost no red dead cells were found in all groups after culturing for 1, 3, and 5 days (Figure 2D). PP group and PLA (the 0:1 PNIPAAm-b-PEG/PLA) group had weaker green fluorescence, indicating less living cells attached to the scaffold. In comparison, the green fluorescence was obviously observed in 1:3, 1:4, 1:5, 1:6, 1:7, and 1:9 scaffolds. Compared with the 1:9 group, the stained live cells were distributed more balanced and the fluorescence intensity was stronger in the 1:5, 1:6, and 1:7 groups.

The cell attachment effects of different scaffolds were examined *via* SEM. A high porosity was observed in the electrospun scaffolds (Figure 2E), and more ADSCs attached to the surfaces of different PNIPAAm-b-PEG/PLA scaffolds with a polygonal shaped spread and significantly extended than to those of the PLA and PP scaffolds indicating a better attachment surface for cells was supplied by the PNIPAAm-b-PEG/PLA biomimetic scaffolds.

Results of biocompatibility confirmed that the 1:7 PNIPAAm-b-PEG/PLA scaffold was biocompatible for further evaluation.

### Hypoxia Upregulated the Expression of HIF-1α in ADSCs

**FIGURE 3:**
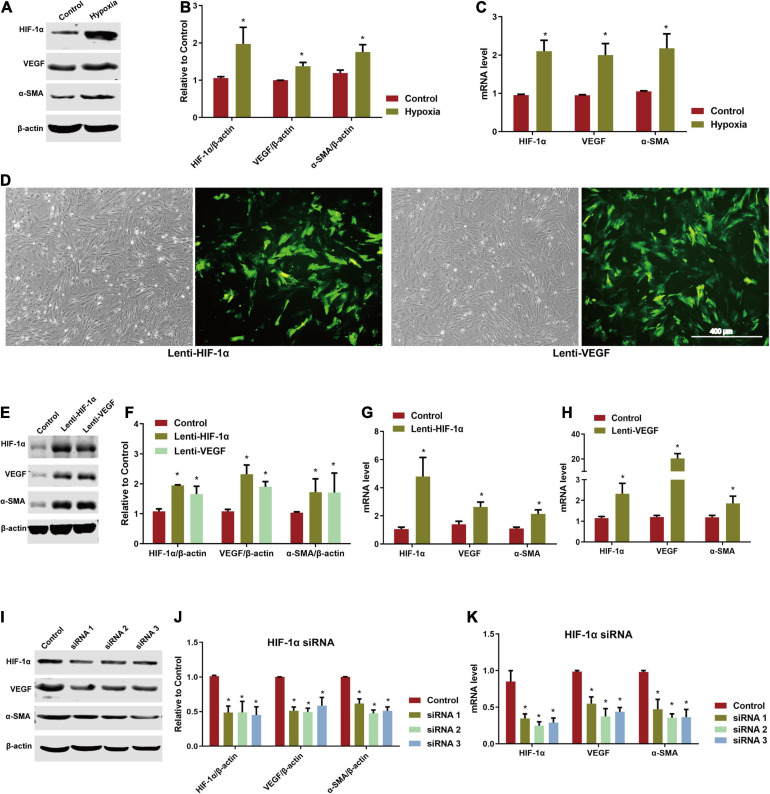
Involvement of HIF-1α/VEGF pathway in directed induction of endothelial differentiation of ADSCs. **(A)** Expression of HIF-1α, VEGF, and α-SMA of hypoxia-induced ADSCs using western blotting. **(B)** Analysis of HIF-1α, VEGF, and α-SMA by western blotting. *P < 0.05 vs. the control (normoxic) group. **(C)** mRNA level of hypoxia-induced HIF-1α, VEGF, and α-SMA in ADSCs by RT-qPCR testing. *P < 0.05 vs. the control (normoxic) group. **(D)** ADSCs transfected by lenti-HIF-1α and lenti-VEGF for 72 h (Scale bar = 400 μm). **(E,F)** Expression and analysis of HIF-1α, VEGF, and α-SMA of lenti-HIF-1α and lenti-VEGF transfected ADSCs by western blotting. *P < 0.05 vs. the control (blank vector) group. **(G,H)** mRNA levels of HIF-1α, VEGF, and α-SMA of lenti-HIF-1α and lenti-VEGF transfected ADSCs by RT-qPCR testing. *P < 0.05 vs. the control (blank vector) group. **(I,J)** Expression and analysis of HIF-1α, VEGF, and α-SMA of ADSCs knocked down by HIF-1α siRNA detected by western blotting. *P < 0.05 vs. the control (negative) group. **(K)** mRNA levels of HIF-1α, VEGF, and α-SMA of ADSCs knocked down by HIF-1α siRNA detected using RT-qPCR. *P < 0.05 vs. the control (negative) group.

In the process of repairing abdominal wall defect, the microenvironment of implanted scaffold area is hypoxic. The expression of HIF and its downstream molecules such as VEGF and α-SMA caused by hypoxia environment is conducive to promoting the endothelialization of ADSCs. Thus, we simulated the hypoxia environment *in vitro*. As showed in Figures 3A–C, HIF-1α expression was significantly increased after 3 weeks of hypoxic culture compared to the control group at normoxia. The expression of VEGF and α-SMA, which was regulated by HIF-1α, was also significant upregulated at the mRNA and protein levels.

### Overexpression of HIF-1α and VEGF Induced Endothelial Differentiation of ADSCs

Adipose-derived stem cells were transfected with lentiviral overexpression systems successfully while fluorescence was obviously observed in both lenti-HIF-1α and lenti-VEGF transfected groups (Figure 3D). The protein and mRNA levels of HIF-1α, VEGF, and α-SMA in the two transfected groups were significantly increased than the control group (blank vector) (Figures 3E–H). When knocking down HIF-1α using siRNA, the protein and mRNA levels of HIF-1α were significantly reduced compared with the negative control group accompanied with the downregulated expression of VEGF and α-SMA (Figures 3I–K). However, cells could not proliferate further after HIF-1α knockdown. After 21 days of culture, WPBs were detected by TEM in the two groups transfected by lenti-HIF-1α and lenti-VEGF indicating the successful induction of ADSCs endothelial differentiation (Supplementary Figure 2).

### PNIPAAm-b-PEG/PLA Biomimetic Scaffold Relieved Abdominal Adhesion

**FIGURE 4:**
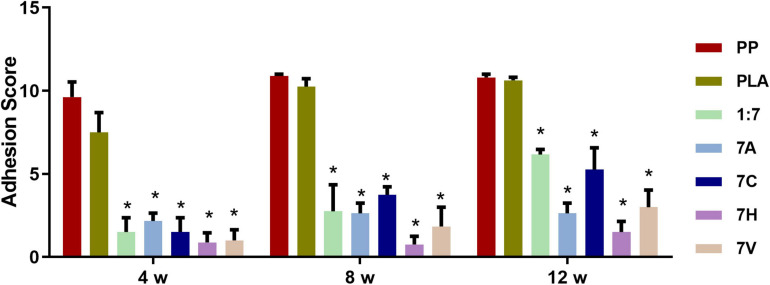
The analysis of abdominal adhesion scores after 4, 8, and 12 weeks of implantation. *P < 0.05 vs. the PP and PLA groups.

The adhesion score was evaluated after 4, 8, and 12 weeks. Results suggested the severe condition of abdominal adhesion in the PP and PLA groups with a high score of 10.9 ± 0.32 and 10.87 ± 0.35 at 12 weeks (Figure 4). The adhesion in these two groups with large vessels, omentums, and opaque intestinal tissue adhered to the defect area was difficult to be separated (Supplementary Figure 3). The adhesion scores in 1:7, 7A (1:7 scaffold seeded with ADSCs), 7C (1:7 scaffold seeded with blank vector transfected ADSCs), 7H (1:7 scaffold seeded with lenti-HIF-1α transfected ADSCs), and 7V (1:7 scaffold seeded with lenti-VEGF transfected ADSCs) groups were significantly lower than the PP and PLA groups. However, there was no significant difference among the five groups.

### Overexpression of HIF-1α and VEGF Promoted Cell Infiltration and Relatively Reduced Collagen Fibrous Deposition

**FIGURE 5:**
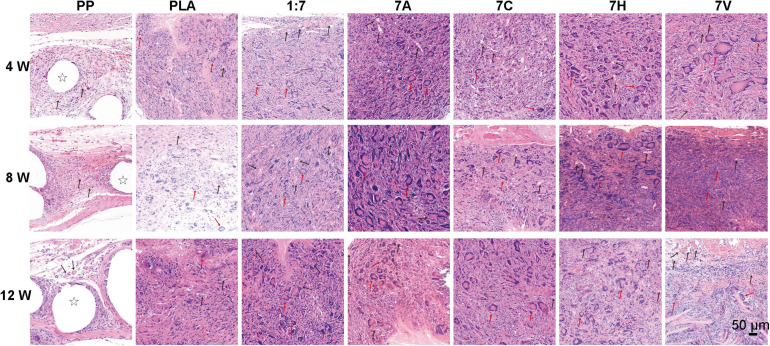
H&E staining used to evaluate histology of scaffolds after 4, 8, and 12 weeks of implantation (Scale bar = 50 μm). Stars indicate PP mesh fibers. Black arrows indicate vessel lumen in regenerative tissue. Red arrows indicate foreign body giant cells in regenerative tissue.

During the period of 12 weeks, the structure of PP mesh was easily identified by H&E staining (Figure 5). However, electrospun scaffolds had already integrated with host cells and tissues. Large numbers of host cells were infiltrated into the six electrospun scaffold groups and rings surrounded by foreign body giant cells (FBGCs) could be observed. In PP group, the regenerative tissue was relatively loose with circular translucent areas showing the mesh structure. Compared with PLA and 1:7 groups, more cells were distributed and infiltrated in 7A, 7C, 7H, and 7V groups. And microvessels were also observed in the regenerative tissue in these four groups.

**FIGURE 6:**
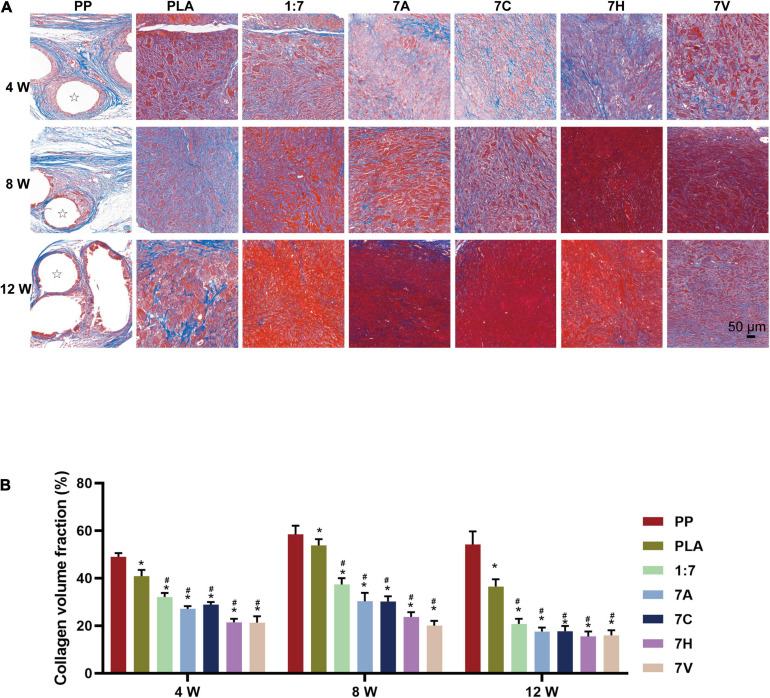
Fibroplasia of regenerative tissue assessed by Masson’s staining. **(A)** Microscopic morphology of regenerative tissue (4, 8, and 12 weeks after implantation, scale bar = 50 μm). Stars indicate PP mesh fibers. **(B)** Collagen volume fraction (CVF) used to quantitative analyze fibroplasia of regenerative tissue (4, 8, and 12 weeks after implantation). *P < 0.05 vs. the PP group, *#P* < 0.05 vs. the PLA group.

In Masson’s staining, collagen fibrous deposition and a few of muscle fibers were observed in PP group with relatively high collagen volume fraction (CVF) of 49.0 ± 3.6%, 58.5 ± 9.0%, and 54.2 ± 13.4% at 4, 8, and 12 weeks indicating a severe fibrosis hyperplasia after implantation of PP mesh (Figures 6A,B). The CVF of PLA group at 4, 8, and 12 weeks were 40.9 ± 6.4%, 53.9 ± 6.3%, and 36.5 ± 7.5%, respectively. Compared with PP and PLA groups, more muscle fibers were generated in other groups after 12 weeks, and the CVF value was significantly lower than 40%. At the 12th week, the CVF value of the 7A, 7C, 7H, and 7V groups seeded with ADSCs showed a decreasing trend compared with the 1:7 group, and the degree of postoperative fibrosis in the regenerative tissue was also reduced. However, no significant difference was found in CVF between 7H and 7V groups.

### Regulation of HIF-1α/VEGF Pathway Facilitated Angiogenesis of Regenerative Tissue

**FIGURE 7:**
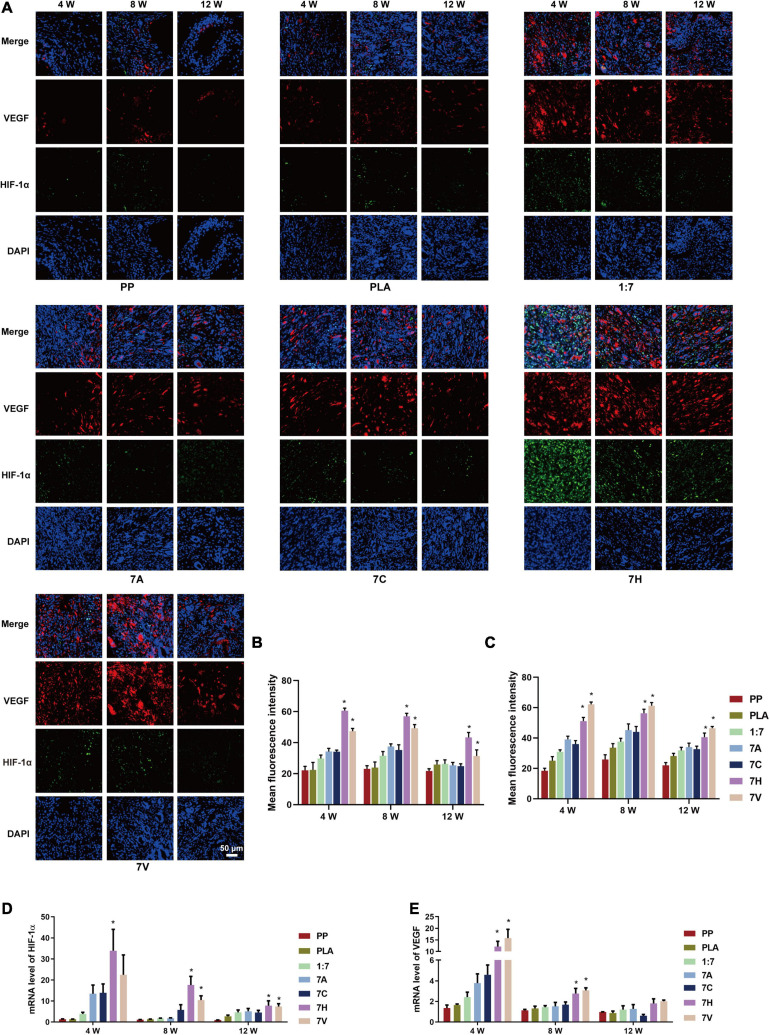
Expression of HIF-1α/VEGF in regenerative tissue of abdominal wall defect model. **(A)** Expression of HIF-1α and VEGF in regenerative tissue detected by immunofluorescent staining (green: HIF-1α, red: VEGF, blue: DAPI) (Scale bar = 50 μm). **(B)** Mean fluorescence intensity of HIF-1α. **(C)** Mean fluorescence intensity of VEGF. **(D,E)** mRNA levels of HIF-1α and VEGF in regenerative tissue detected by RT-PCR testing. *P < 0.05 vs. the PP, PLA, 1:7, 7A, and 7C groups.

Compared with other groups, HIF-1α stained green was strongly positive in the 7H group by immunofluorescent staining (Figure 7A). VEGF stained red was found to be overexpressed in the groups, except PP and PLA groups. Further results indicated VEGF was strongly positively expressed in the 7H and 7V groups. The fluorescence intensity and mRNA level of HIF-1α in 7H group reached the peak value (60.65 ± 5.36 and 33.87 ± 30.51, respectively) at 4 weeks, but decreased gradually in the following 8 weeks (Figures 7A,B,D). Overexpression of VEGF in 7H and 7V sustained 12 weeks (Figures 7A,C,E). The results of mRNA further showed HIF-1α and VEGF were more expressed in 7H and 7V than in other groups; however, there was no difference in the mRNA expression of the two targets between 7H and 7V at 12 weeks (Figures 7D,E).

**FIGURE 8:**
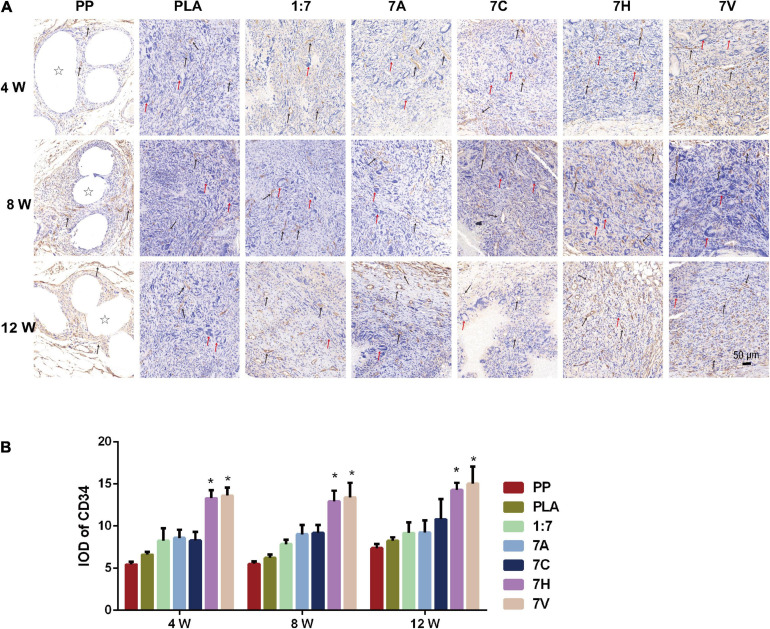
CD34 expression in regenerative tissue detected and analyzed by immunohistochemistry. **(A)** Microscopic morphology of regenerative tissue (Scale bar = 50 μm). Stars indicate PP mesh fibers. Black arrows indicate vessel lumen and CD34 expression in regenerative tissue. Red arrows indicate foreign body giant cells in regenerative tissue. **(B)** IOD was used to analyze the expression of CD34. *P < 0.05 vs. the PP, PLA, 1:7, 7A, and 7C groups.

Immunohistochemical detection of CD34 in regenerative tissue showed positive expression in all groups (Figure 8). At fourth week, fewer of vascular lumen tissue were observed in the PP group while there were more in the PLA group. But, the average optical density (IOD) between the two groups was 5.45 ± 0.99 and 6.60 ± 1.00 with no significant difference. The expression of CD34 found in 7H and 7V groups was significant higher than that of the other five groups during the 12 weeks. However, the IOD of 7H group was not significantly increased compared with that of 7V. Moreover, more smooth muscles marked by α-SMA were generated in the 7H and 7V groups than other groups, which was detected by immunohistochemical staining and RT-qPCR testing at the mRNA level (Supplementary Figure 4).

**FIGURE 9:**
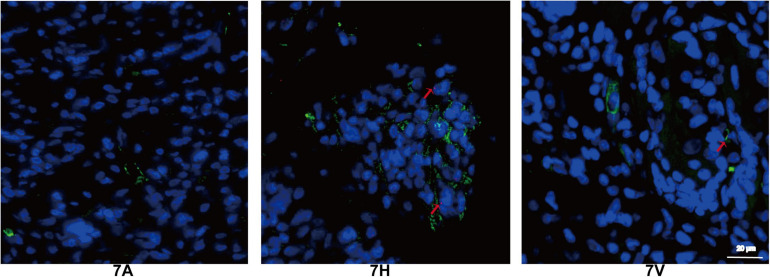
Tracking of ADSCs in regenerative tissue by FISH at 12 weeks (green: CD34, orange: Y chromosome, blue: DAPI). Red arrows indicate endothelial cells differentiated from ADSCs (Scale bar = 20 μm).

Furthermore, endothelial cells were detected by CD34 and Y chromosome paint probe staining in 7A, 7H, and 7V groups (Figure 9) at 12th week. In the female rat model of abdominal wall defect, the double labeled cells found in the 7H and 7V groups indicated endothelial cells were differentiated from the seeded male ADSCs though the endothelial differentiation rate was low.

## Discussion

Tissue engineering scaffolds provide a prospective method for the repair and regeneration of damaged tissues and organs by combining materials science and cell biology (Langer and Vacanti, 1993). Tissue regeneration involves seed cell integration, scaffolds, and appropriate biochemical and physio-chemical factors to promote tissue growth. ADSCs isolated from adipose tissues are one of the most promising seed cells for the reconstruction of soft tissue damage, including abdominal wall defects with multidirectional differentiation potential (Naderi et al., 2017). ADSCs have the advantages of easy extraction, abundant sources, and minor damage to the donor compared to those by skeletal muscle stem cells, bone marrow mesenchymal stem cells, and other tissue-derived stem cells (Zhan et al., 2016). The results of flow cytometry and differentiation indicated ADSCs isolated from rat had high purity and multi-lineage differentiation, and were appropriate for tissue engineering.

Scaffolds fabricated using electrospun nanofibers have been explored in many biomedical applications, such as tissue engineering and regenerative medicine, for their ability to simulate the natural extracellular matrix (Xue et al., 2017). Good biocompatibility, biodegradation, promoting cell adhesion and proliferation, harmless to cell physiological activities, and sufficient mechanical strength are the basic characteristics of ideal electrospinning tissue engineering scaffolds (Hall Barrientos et al., 2019; Doostmohammadi et al., 2020). Material is one of the significant factors determining the biological characteristics of scaffolds. The key to fabricating a scaffold is to find a non-toxic, biodegradable and biocompatible material (Hong et al., 2019). PNIPAAm-b-PEG, a block polymer of PNIPAAm, had an improved degradation capability and was beneficial for cell adherence and proliferation (Pollock and Healy, 2010). It was certain that scaffolds consisted of PNIPAAm-b-PEG had the potential to promote cell infiltration and viability. Improving the mechanical strength of electrospun scaffolds to satisfy the physiological need of the abdominal wall is still a challenge (Xue et al., 2017). It was reported the minimum physiological stiffness of the abdominal wall was 1.6 N/mm while the stiffness should be at least 2.0 N/mm considering some extreme behaviors (Mazza and Ehret, 2015). With the involvement of PLA, the PNIPAAm-b-PEG/PLA scaffolds enhanced the mechanical strength and were consequently suitable for applications *in vivo*.

Electrospun scaffolds stimulated the native extracellular matrix formation and offered appropriate pores to steadily maintain biological fluids for cell adhesion and proliferation (Pollock and Healy, 2010). With the help of physical blending method, electrospun nanofibers fabricated by thermoresponsive hydrogel and another polymer could retain thermosensitive property to achieve drug controlled release (Gu et al., 2010; Lin et al., 2012). Thus, the EWC variation of PNIPAAm-b-PEG/PLA scaffold was attributed to the hydrogel properties of PNIPAAm-b-PEG. PNIPAAm-b-PEG/PLA scaffold with high EWC has large capacity to maintain abundant fluids and nutriments, contributing to cell proliferation and migration in the repair region. Water contact angle is an important parameter which determined the hydrophobic or hydrophilic property of material interface. It is hydrophobic when the angle exceeds 90° (Ma et al., 2007). The water contact angle indicated that PNIPAAm-b-PEG could facilitate the hydrophilicity of biomimetic scaffold and make it easy for cell adhesion. The controlled degradation rate is also significant for PNIPAAm-b-PEG/PLA scaffold which was observed by visual FBGCs in histology and probably influenced by PNIPAAm-b-PEG.

Biocompatibility was another significant characteristic. The test of material toxicity plays an important role in the safety assessment of the implanted scaffolds. The results revealed PNIPAAm-b-PEG had a beneficial effect on the ADSC proliferation without notable toxicity. The cytocompatibility results were consistent with the non-toxic properties of PNIPAAm-b-PEG/PLA scaffolds, which demonstrated the improved characteristics of scaffold’s surface for easy cell adhesion and proliferation by PNIPAAm-b-PEG.

Hypoxia in abdominal wall reconstruction resulting from a limited oxygen diffusion distance (150–200 μm) *in vivo*, low blood supply in the implanted material, and increased tissue oxygen demand in the reconstruction area impeded tissue repair and regeneration (Staubli et al., 2017). Early vascularization is the key component to reducing negative influence of anoxia. ADSCs possessed various potentials as types of ideal seed cells in tissue engineering, differentiating into adipocytes, osteoblasts, and endothelial cells (Naderi et al., 2017). Studies have reported that ADSCs seeded in tissue scaffolds can accelerate the repair of vessel, bone, and nerve (Wang and Liu, 2018). Hence, early vascularization could be achieved by inducing endothelial differentiation of ADSCs. Though various methods have been used to induce ADSCs differentiating into endotheliocytes *via* upregulating VEGF, it is still limited by certain problems, such as imbalanced cytokine distribution, short half-life of the biological activity, and uncontrolled release (Volz et al., 2016; Ma et al., 2017; Chen and Liao, 2018). Compared with exogenous addition of cytokines or pretreatment *in vitro*, target genes transfected into ADSCs using lentiviral overexpression technology can be more precise to regulate the long-term expression of proteins such as VEGF (Gouvarchin Ghaleh et al., 2020). The transfected technology is commonly utilized within the growing field of cell and gene therapy for the treatment of monogenic diseases and adoptive therapies (Perry and Rayat, 2021). Therefore, genes transfected method was applied for inducing endothelial differentiation of ADSCs in our study.

HIF-1α is another viral transcription factor in the process of early vascularization, whereas its abnormal expression in abdominal wall defects leads to severe disruption of tissue regeneration. HIF-1α is stable in hypoxic condition which can regulate the cytokine secretion of ADSCs (Lang et al., 2019). Upregulated expression of HIF-1α improved the vascularized effect of the stem cells, whereas silencing HIF-1α caused continuous wound non-healing (Hong et al., 2014). In addition, cells or animals can die after knocking out HIF-1α (Lang et al., 2019). Our study showed short-term hypoxia simulating the early period of abdominal wall defect area increased the expression of HIF-1α and its target genes VEGF and α-SMA. Cells including ADSCs, endothelial cells, fibroblasts, and inflammatory cells in regenerative tissue could also express HIF-1α in anoxic state (Ball et al., 2012). The interdependent regulation between HIF-1α and VEGF in ADSCs could increase HIF-1α expression in 7H and 7V groups which affected the expression of downstream target genes and further promoted the migration, adhesion and cytokine secretion of host cells (Kunugiza et al., 2010; Ruthenborg et al., 2014; Wang et al., 2017; Du et al., 2018). Host cells migrated and adhered to the regenerative area increased oxygen demand and led to local regional hypoxia maintaining the stability and increase of HIF-1α (Lokmic et al., 2012). In this condition, HIF-1α and its related pathways could play positive effects on tissue repair and regeneration (Ruthenborg et al., 2014). However, persistent hypoxia inhibited cell metabolism, leading to a significant decreased expression of HIF-1α and its downstream genes, such as VEGF and platelet derived growth factor, involved in angiogenesis (Ruthenborg et al., 2014; Eskandani et al., 2017; Desmet et al., 2018). Thus, it was necessary to realize vascularization in the early period of repair and regeneration.

It was reported increased HIF-1α and VEGF expression in ADSCs significantly promoted angiogenesis and functional recovery in the myocardial infarction in animal models (Wang et al., 2017). Besides, tissue repair could be enhanced by regulating HIF-1α and its downstream genes to promote host cell migration and adhesion, and also stimulate angiogenesis and muscular tissue formation in wound repair (Ruthenborg et al., 2014). In our study, endothelial differentiation of ADSCs was induced by overexpression of HIF-1α or VEGF which was identified by presentation of endothelial cell marker WPBs. What’s more, overexpression of HIF-1α stimulated more endothelial cells and related muscular tissue formation, relieved abdominal adhesion, relatively slowed down hyperplasia of collagen fibers in the regenerated tissues, and consequently stimulated angiogenesis in the scaffolds. A similar phenomenon was observed when the expression of VEGF was increased. Analysis of CD34 and Y chromosome paint probe staining yielded a low ratio of differentiation. The possible reasons might include the low rate of differentiating ADSCs into endothelial cells, their limited life span, and the immersion and proliferation of host cells leading to the dilution of exogenous stem cells (Han et al., 2015; Kim et al., 2016). However, this did not mean that the capacity of stem cells to promote vascularization and tissue regeneration is low. Upregulation of the HIF-1α and VEGF resulted in enhanced early vascularization in the biomimetic scaffolds. These conditions revealed HIF-1α/VEGF pathway play a vital role in angiogenesis in the repair and regeneration of abdominal wall defects.

## Conclusion

Summarizing this study, a biocompatible scaffold was obtained consisting of a thermoresponsive gel, PNIPAAm-b-PEG, and a degradable polymer, PLA, when electrospun in a mass ratio of 1:7. ADSCs were successfully seeded in the biomimetic scaffold before implantation in the abdominal wall defect. By regulating the HIF-1α/VEGF pathway, the endothelial differentiation of ADSCs was induced and early vascularization was realized. Overall, our study showed that the regeneration and repair of abdominal wall defects could be facilitated by the endothelial differentiation of ADSCs based on a biomimetic scaffold, which may lead to beneficial development.

## Data Availability Statement

The raw data supporting the conclusions of this article will be made available by the authors, without undue reservation.

## Ethics Statement

The animal study was reviewed and approved by the Animal Experimental Ethics Committee of Shanghai Ninth People’s Hospital affiliated to the Shanghai Jiao Tong University School of Medicine.

## Author Contributions

All authors listed have made a substantial, direct and intellectual contribution to the work, and approved it for publication.

## Conflict of Interest

The authors declare that the research was conducted in the absence of any commercial or financial relationships that could be construed as a potential conflict of interest.
