# Supplementary material for: Adipose-Derived Stem Cells Based on Electrospun Biomimetic Scaffold Mediated Endothelial Differentiation Facilitating Regeneration and Repair of Abdominal Wall Defects via HIF-1α/VEGF Pathway
Source: Front Bioeng Biotechnol. 2021 Jul 7;9:676409. doi: 10.3389/fbioe.2021.676409 (PMC8293919; doi:10.3389/fbioe.2021.676409)
Supplement: Supplementary file 1 [file Data_Sheet_1.docx]

Supplementary Material

# Supplementary Figures

**Supplementary Table 1**. The marking criterion of abdominal adhesions(Poehnert et al., 2015).

| Adhesion characteristics | Score |
| --- | --- |
| Extent of site involvement |  |
| None | 0 |
| < 25% | 1 |
| < 50% | 2 |
| < 75% | 3 |
| < 100% | 4 |
| Type |  |
| None | 0 |
| Filmy, transparent, avascular | 1 |
| Opaque, translucent, avascular | 2 |
| Opaque, capillaries present | 3 |
| Opaque, large vessels present | 4 |
| Tenacity |  |
| None | 0 |
| Adhesion falls apart | 1 |
| Adhesion lysed with traction | 2 |
| Adhesion requiring sharp dissection | 3 |
| Total | 11 |


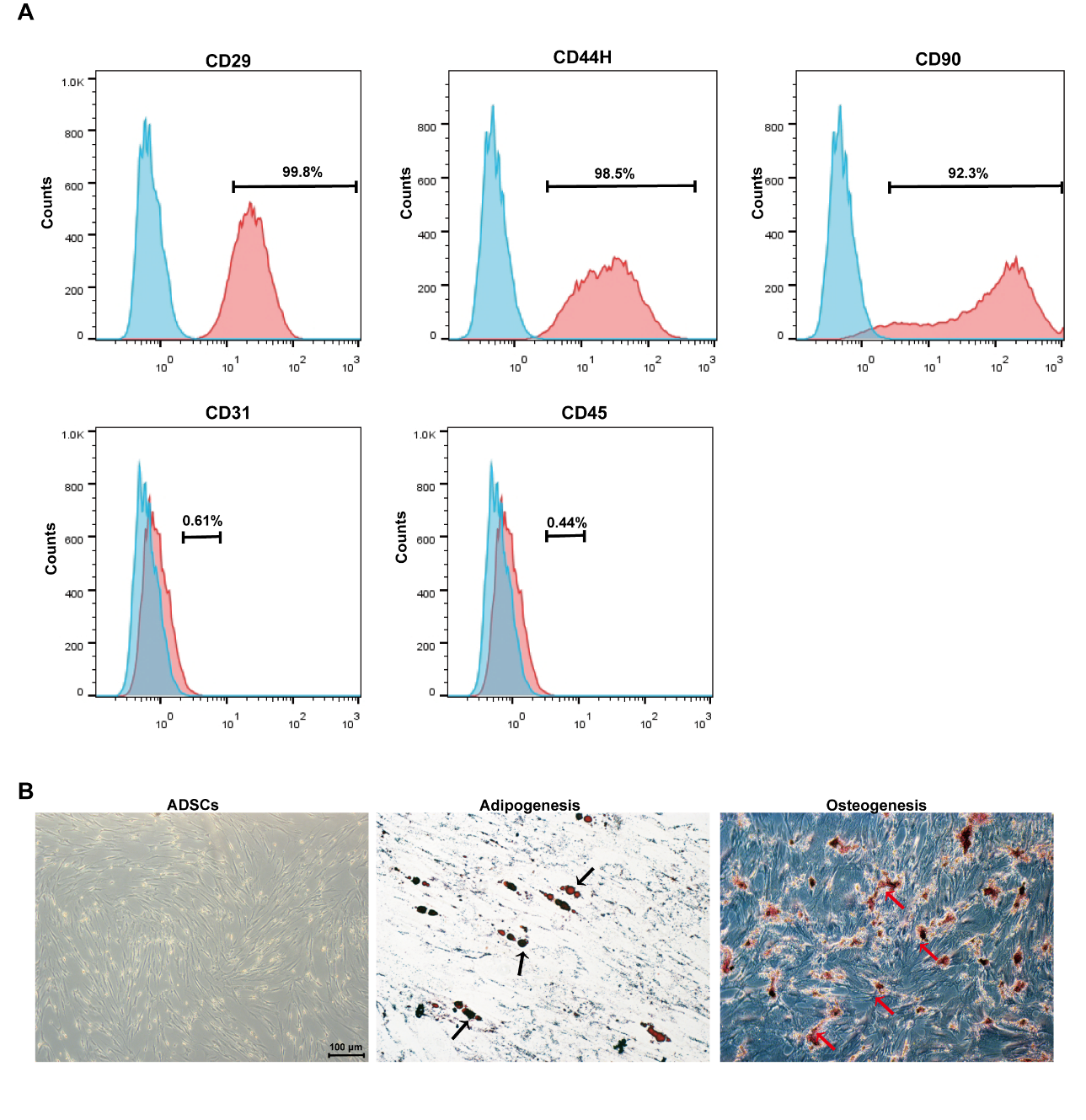


**Supplementary Figure 1.** Characterisation of ADSCs. (A) Flow cytometry was used to detect cell markers; it confirmed positive expression of CD29, CD44H, and CD90 in rat ADSCs, with negative expression of CD31 and CD45. (B) Multi-differentiation potential was identified by adipogenic and osteogenic differentiation. Adipogenic differentiation was tested by oil red-O staining with intracellular lipid (black arrows) compared with the control ADSCs, whereas osteogenic differentiation was assessed by alizarin red staining with intracellular calcium deposits (red arrows) stained in red (Scale bar = 100 μm).


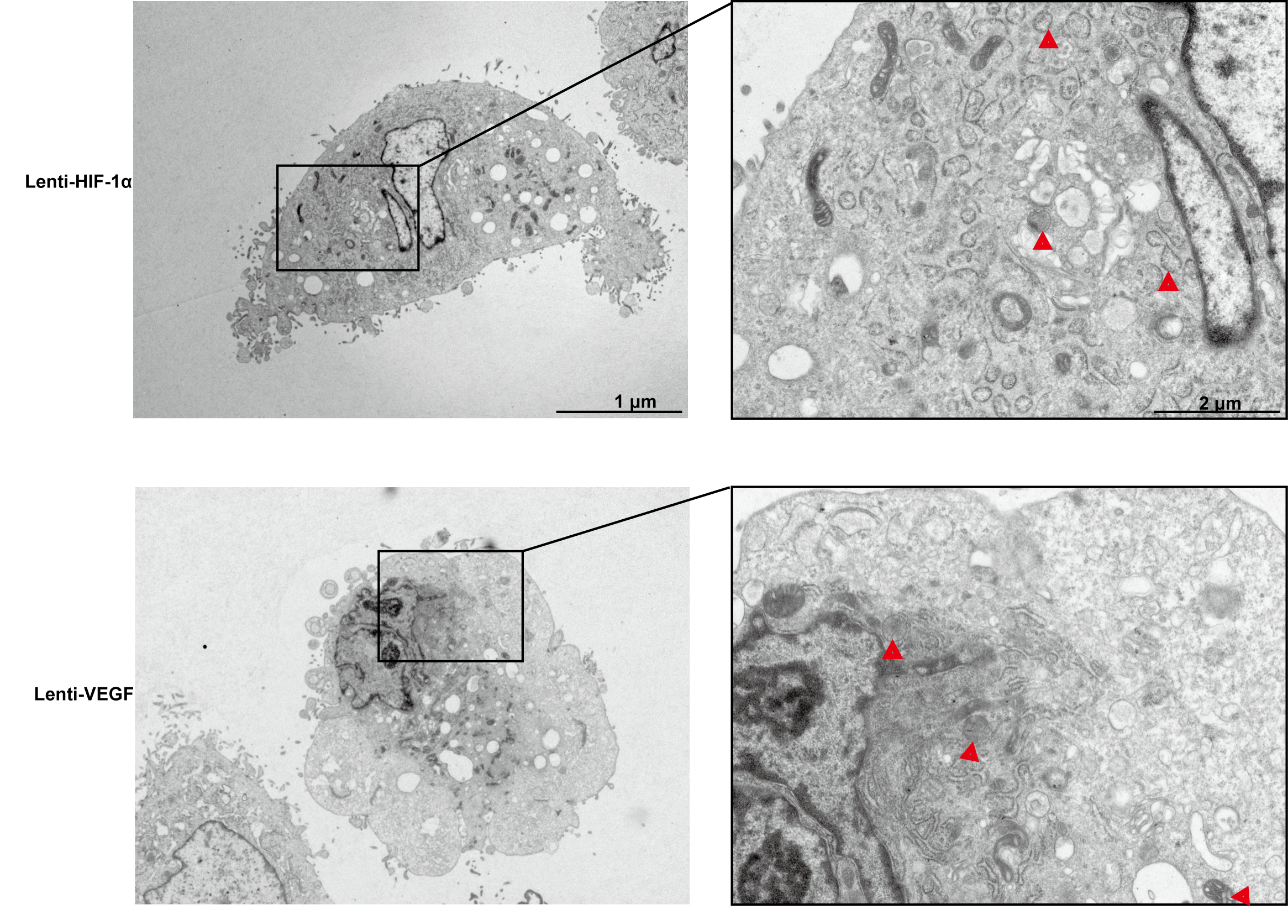
**Supplementary Figure 2.** WPBs (red triangles) observed in lenti-HIF-1α and lenti-VEGF transfected ADSCs via TEM.


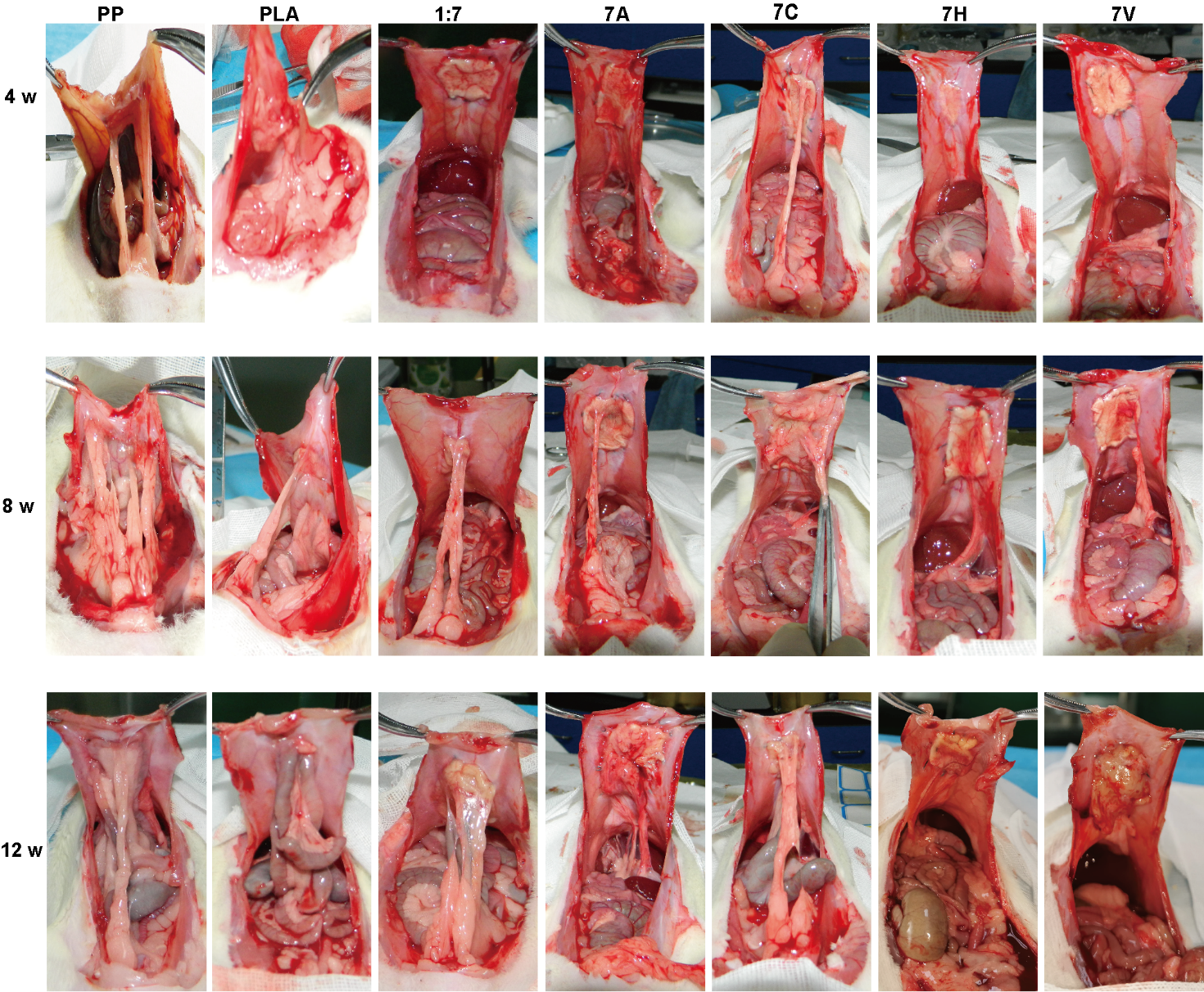


**Supplementary Figure 3.** Gross view of abdominal adhesion after 4, 8, and 12 weeks of implantation.

**
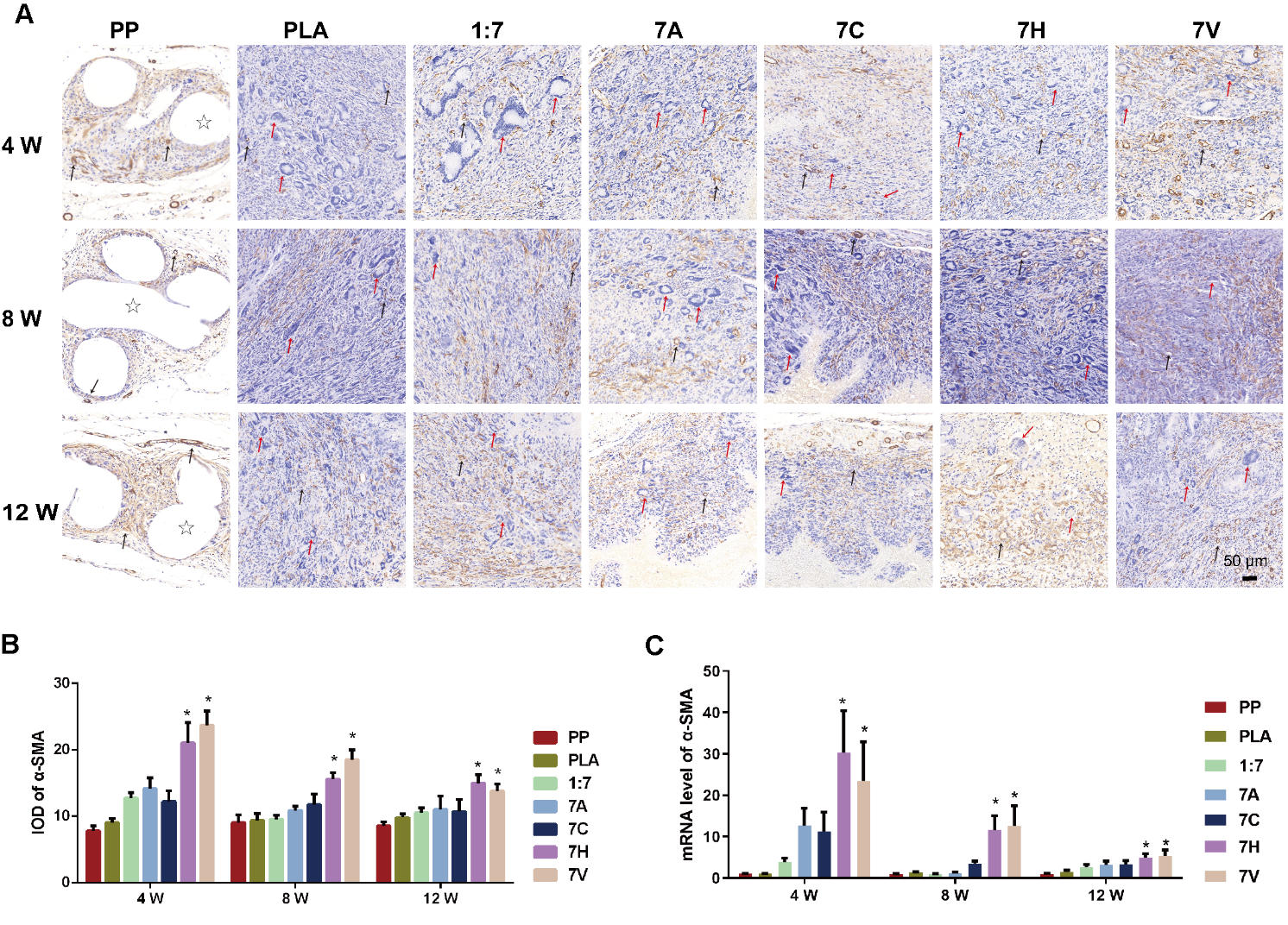
**

**Supplementary Figure 4.** α-SMA expression in regenerative tissue of abdominal wall defect model detected and analyzed by immunohistochemistry. (A) Microscopic morphology of regenerative tissue (Scale bar = 50 μm). Stars indicate PP mesh fibres. Black arrows indicate α-SMA expression in regenerative tissue. Red arrows indicate foreign body giant cells in regenerative tissue. (B) IOD was used to analyze the expression of α-SMA. (C) mRNA levels of α-SMA detected using RT-PCR. **P* < 0.05 vs the PP, PLA, 1:7, 7A, and 7C groups.

# REFERENCES

Poehnert, D., Abbas, M., Kreipe, H.H., Klempnauer, J., and Winny, M. (2015). High reproducibility of adhesion formation in rat with meso-stitch approximation of injured cecum and abdominal wall. *Int J Med Sci* 12(1)**,** 1-6. doi: 10.7150/ijms.8870.
